# Newly Identified Function of Caspase-6 in ZBP1-mediated Innate Immune Responses, NLRP3 Inflammasome Activation, PANoptosis, and Host Defense

**DOI:** 10.33696/immunology.2.064

**Published:** 2020

**Authors:** Min Zheng, Thirumala-Devi Kanneganti

**Affiliations:** Department of Immunology, St. Jude Children’s Research Hospital, Memphis, TN, 38105, USA

**Keywords:** Caspase-6, ZBP1, Influenza A virus, Innate immunity, NLRP3, Inflammasome, RIPK3, RIPK1, Caspase-1, Caspase-8, Pyroptosis, Apoptosis, Necroptosis, PANoptosis, PANoptosome

## Abstract

Caspase-6 was discovered decades ago, but its roles in biological processes remain largely unknown. Recently, we have demonstrated that caspase-6 plays a critical role in influenza A virus (IAV)-induced cell death and innate immune responses. During IAV infection, Z-DNA binding protein 1 (ZBP1) initiates ZBP1-PANoptosome assembly to drive inflammasome activation and cell death, and we showed that caspase-6 interacts with RIPK3 to enhance the interaction between RIPK3 and ZBP1, thus promoting PANoptosome assembly. Moreover, the caspase activity of caspase-6 is not required for tins process, suggesting a caspase-independent function of caspase-6 during IAV infection. Additionally, we found that caspase-6 is required for the alternative activation of alveolar macrophages in response to IAV infection. Our findings provide an opportunity to reconsider the physiological role of caspase-6.

## Caspase-6: An Apoptotic and Non-apoptotic Caspase

Caspases are critical for regulating cell death, immune responses, and homeostasis. These cysteine-dependent endoproteases cleave their substrates after certain aspartic acid residues [1]. So far, thirteen caspases have been discovered in mice and humans. They are composed of an N-terminal pro-domain of variable size and a C-terminal protease domain consisting of one small and one large catalytic subunit [2,3]. Caspases have been found to be involved in several biological processes, including inflammatory responses, programmed cell death, homeostasis, and cell differentiation [2,4]. Those that directly participate in the inflammatory cell death pathways are called inflammatory caspases, including caspase-1, -4, -5, and -11, while those mediating apoptosis activation are known as apoptotic caspases, including caspase-3, -6, -7, -8, -9, and -10 [2].

Apoptotic caspases can be classified as initiator caspases (caspase-8, -9, and -10) and executioner caspases (caspase-3, -6, and -7) [2]. Caspase-6 has long been recognized as an apoptotic caspase. Overexpression of caspase-6 can induce apoptosis in mammalian cells [5]. Additionally, it can mediate nuclear apoptosis after activation by processing Lamin A/C, caspase-6–specific nuclear intermediate filament proteins, and nuclear mitotic apparatus protein [6–8]. Very recently, one report showed that caspase-6–induced apoptosis is a key step for the development of nonalcoholic steatohepatitis [9]. However, the full mechanistic details of how caspase-6 is activated remain unclear. Reports suggest that it can be activated in both a caspase-3–dependent and –independent manner [10–13]. Also, caspase-6 can undergo autoactivation both *in vitro* and *in vivo* [14,15]. It is possible that the activation of caspase-6 is a context-dependent event. More studies are required to determine the detailed mechanism of caspase-6 activation.

In addition to being an executioner of apoptosis, caspase-6 can also act as a non-apoptotic caspase. Caspase-6 plays crucial roles in several neurodegenerative diseases, including Huntington’s disease [16–19], Alzheimer’s disease [20–22], and Parkinson’s disease [23]. While active caspase-6 has been detected in these processes, its activation does not lead to apoptosis in these contexts. Additionally, caspase-6 has also been found to be involved in several inflammatory responses. Caspase-6 can cleave IL-1 receptor-associated kinase M to regulate neutrophil-mediated TNF production in macrophages [24]. Another study reported that active caspase-6 can induce microglial TNF secretion to drive inflammatory pain [25]. Furthermore, caspase-6 was shown to be critical for the differentiation of alternatively activated macrophages [26]. All these studies indicate that caspase-6 has dual functions, acting as both an apoptotic and non-apoptotic caspase.

## Caspase-6 in Innate Immunity and Host Defense

Despite the classification of caspase-6 as an apoptotic caspase and the knowledge of its association with neurological disorders, the functions and importance of caspase-6 in innate immunity have remained largely a mystery. In our recent study, we found that caspase-6 is critical for influenza A virus (IAV)-induced cell death and inflammatory responses [27]. In the absence of caspase-6, IAV-induced cell death and inflammasome activation is attenuated in bone marrow-derived macrophages (BMDMs). Similarly, the amount of inflammasome-related cytokines, IL-1β and IL-18, is significantly reduced in the supernatants of IAV-infected *Casp6*^−/−^ cells. Furthermore, caspase-6 was critically involved in the host defense against IAV infection in a murine model, and *Casp6*^−/−^ mice were more likely to succumb to infection and had a higher viral burden [27].

## Caspase-6 in PANoptosis

**Figure 1:**
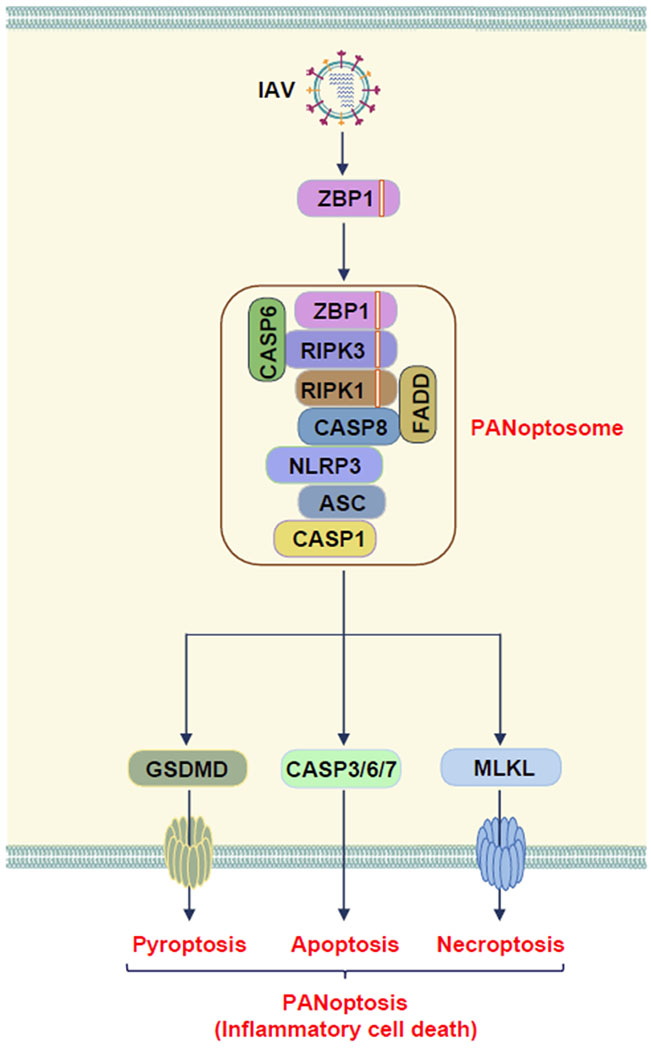
IAV induces PANoptosis through the formation of the ZBP1-PANoptosome.

During IAV infection, Z-DNA-binding protein 1 (ZBP1) acts as an innate immune sensor to detect IAV vRNPs and activate cell death through PANoptosis [27–33]. PANoptosis (‘P’, Pyroptosis; ‘A’, Apoptosis; ‘N’, Necroptosis; and ‘optosis’) is defined as “a unique inflammatory programmed cell death regulated by the PANoptosome, which provides a molecular scaffold that allows for the interactions and activation of machinery required for inflammasome/pyroptosis (such as NLRP3, ASC, caspase-1), apoptosis (caspase-8), and necroptosis (RIPK3/RIPK1)” [27,31,32,34]. The ability of these molecules to interact allows for intricate coregulation between cell death pathways that had previously been thought to be independent. PANoptosis has been implicated in infectious and autoinflammatory disease, cancer, and beyond [27,28,31,34–39]. We found that caspase-6 is a critical component of the ZBP1-PANoptosome (Figure 1) [27]. Without caspase-6, both PANoptosis and inflammasome activation following IAV infection are impaired, suggesting the essential role of caspase-6 in the assembly of the PANoptosome.

Mechanistically, ZBP1 contains two nucleic acid sensing Zα domains and a RIP homotypic interaction motif (RHIM). The Zα2 domain is critical for sensing IAV upstream of PANoptosis [28,40–42]. The RHIM then allows ZBP1 to form a complex with RIPK3 and RIPK1, two other RHIM-containing proteins, to drive cell death [28,40–42]. In this process, caspase-6 interacts with RIPK3, thus enhancing the interaction between RIPK3 and ZBP1 in the presence of RIPK1 [27]. It has been shown that without a functional RIPK1 RHIM domain, ZBP1 can sense endogenous ligands and interact more strongly with RIPK3 to activate necroptosis [29,43–46], suggesting that RIPK1 competes with RIPK3 to interact with ZBP1. Our study provides a possible explanation for how ZBP1 interacts with RIPK3 in the presence of RIPK1 under physiological conditions. Through binding to caspase-6, the affinity of RIPK3 for ZBP1 is increased during IAV infection, without altering the binding activity of RIPK1. However, it is unclear how and when caspase-6 binds to RIPK3 in this process. Also, more studies are needed to test the requirement of caspase-6 in other biological processes that can recruit RIPK3 to ZBP1. It is clear that RIPK3 acts as a key adaptor for the ZBP1-initiated PANoptosome [28,41,42]. However, in the absence of RIPK3, ZBP1-mediated, IAV-induced cell death is increased later in the infection phase, and this cell death signal is transmitted through RIPK1 [42]. This implies that the components of the IAV induced-PANoptosome may vary over time throughout the progression of the infection. More investigations are warranted to elucidate the dynamics of the ZBP1-PANoptosome components and how caspase-6 is involved in these cell death mechanisms.

## Caspase Activity-independent Function of Caspase-6

It is interesting to note that the caspase activity of caspase-6 is not required for it to enhance the interaction between RIPK3 and ZBP1, evidenced by the fact that both caspase-dead or uncleavable mutants of caspase-6 can interact with RIPK3 and therefore promote the interaction between ZBP1 and RIPK3. Additionally, both the N- and C-terminal domains of caspase-6 can immunoprecipitate the full length, N-, and C-terminal domains of RIPK3. Likewise, both N- and C-terminal domains of caspase-6 have the potential to increase the binding of RIPK3 to ZBP1. Since both caspase-6 and RIPK3 contain intrinsically disordered regions [27,47,48], it is likely that the interaction between caspase-6 and RIPK3 is mediated by those regions. Overall, our study suggests that caspase-6 possesses a caspase-independent role in cell death. This is unique from its role in neurodegenerative diseases or other inflammatory responses, during which the catalytic activity of caspase-6 is required for the proteolytic activation of its substrates to execute its non-apoptotic function [18,19,21–26]. Our study is the first to show that caspase-6 can function without caspase activity [27].

Caspase-6 is not the only caspase that possesses caspase-independent functions. Caspase-8 has been shown to act as a scaffold to promote inflammasome activation downstream of Toll-like receptors [49] and proinflammatory cytokine production induced by TRAIL [50]. Recently, the scaffold function of caspase-8 has also been shown to be important in development. In mice expressing a caspase-dead version of caspase-8, deletion of MLKL leads to apoptosis and pyroptosis in the intestines, suggesting that caspase-8 can act as a scaffold to promote cell death during development [51,52]. These studies suggest that caspase proteins have the potential to play caspase-independent roles in biological processes. It is therefore not unprecedented to find that caspase-6 could regulate the RHIM-mediated interaction between RIPK3 and ZBP1 via binding to RIPK3 in response to IAV infection in the absence of caspase activity. It is important to note, however, that due to the limitation of currently available caspase-6 antibodies, we did not detect an endogenous interaction between RIPK3 and caspase-6 in primary cells. We did find that the binding of ZBP1 to RIPK3 is reduced during IAV infection in caspase-6–deficient primary cells. Developing better caspase-6 antibodies may help resolve this issue. Also, the generation of transgenic mice with tagged caspase-6 is another promising method that could be used to confirm the endogenous protein interactions. Additionally, RHIM-mediated interactions tend to form a detergent insoluble amyloid-like complex [48,53–56]. It is possible that caspase-6 interacting with RIPK3 is present in the insoluble part of cell lysates under physiological conditions. More studies are required to clarify these potential mechanisms.

## Perspectives

Although it was discovered decades ago, caspase-6 has remained enigmatic. As an apoptotic executioner caspase, it seems to be redundant with caspase-3 and -7. Initial studies demonstrated that caspase-6 is critical for nuclear apoptosis [8,57], but studies with *Casp6*^−/−^ cells indicate that nuclear breakdown is normal in the absence of caspase-6 during apoptosis [58]. Additionally, deleting caspase-3 and -7 can block both extrinsic and intrinsic apoptosis in human leukemia cells, but deleting caspase-3/-6 or caspase-6/-7 does not have any inhibitory effect on extrinsic or intrinsic apoptosis [59]. These studies indicate that caspase-6 is dispensable during apoptosis and that it can be compensated by the other proteases, which raises a question about its physiological role. It is possible that the primary function of caspase-6 is as a non-apoptotic caspase. It will be informative to conduct mass spectrometry analyses to identify the interactome of caspase-dead or uncleavable caspase-6. Given that the currently available antibodies for mouse caspase-6 do not have high enough affinity and specificity for immunoprecipitation, it will be important to establish transgenic mice expressing tagged mutants of caspase-6 and use primary cells or tissue samples to conduct immunoprecipitations. Similar strategies can be employed to investigate the PANoptosome components that are induced by IAV throughout the timeline of infection to study the role of caspase-6 and other molecules at different infection phases.

Overall, beyond its originally identified apoptotic functions, caspase-6 has critical roles in innate immunity, ZBP1-mediated NLRP3 inflammasome activation, PANoptosis, and host defense during IAV infection. While the molecular details of the function of caspase-6 in the PANoptosome have been described, this caspase still has many unsolved mysteries to be investigated.

## References

[1] Lamkanfi M, Declercq W, Kalai M, Saelens X, Vandenabeele P. Alice in caspase land. A phylogenetic analysis of caspases from worm to man. Cell Death Differ. 2002 4;9(4):358–61. 11965488 10.1038/sj.cdd.4400989

[2] Van Opdenbosch N, Lamkanfi M. Caspases in Cell Death, Inflammation, and Disease. Immunity. 2019 6 18;50(6):1352–64. 31216460 10.1016/j.immuni.2019.05.020 PMC6611727

[3] Man SM, Kanneganti TD. Converging roles of caspases in inflammasome activation, cell death and innate immunity. Nat Rev Immunol. 2016 1;16(1):7–21. 26655628 10.1038/nri.2015.7 PMC4915362

[4] Galluzzi L, Lopez-Soto A, Kumar S, Kroemer G. Caspases Connect Cell-Death Signaling to Organismal Homeostasis. Immunity. 2016 2 16;44(2):221–31. 26885855 10.1016/j.immuni.2016.01.020

[5] Suzuki A, Kusakai G-I, Kishimoto A, Shimojo Y, Miyamoto S, Ogura T, Regulation of caspase-6 and FLIP by the AMPK family member ARK5. Oncogene. 2004 9 16;23(42):7067–75. 15273717 10.1038/sj.onc.1207963

[6] Hirata H, Takahashi A, Kobayashi S, Yonehara S, Sawai H, Okazaki T, Caspases are activated in a branched protease cascade and control distinct downstream processes in Fas-induced apoptosis. J Exp Med. 1998 2 16;17(4):587–600. 10.1084/jem.187.4.587 PMC2212161 9463409

[7] Orth K, Chinnaiyan AM, Garg M, Froelich CJ, Dixit VM. The CED-3/ICE-like protease Mch2 is activated during apoptosis and cleaves the death substrate lamin A. J Biol Chem. 1996 7 12;271(28):16443–6. 8663580

[8] Ruchaud S, Korfali N, Villa P, Kottke TJ, Dingwall C, Kaufmann SH, Caspase-6 gene disruption reveals a requirement for lamin A cleavage in apoptotic chromatin condensation. EMBO J. 2002 4 15;21(8):1907–77. 10.1093/emboj/21.8.1967 PMC125972 11953316

[9] Zhao P, Sun X, Chaggan C, Liao Z, Wong K in, He F, An AMPK–caspase-6 axis controls liver damage in nonalcoholic steatohepatitis. Science. 2020 2 7;367(6478):652–60. 32029622 10.1126/science.aay0542 PMC8012106

[10] Slee EA, Harte MT, Kluck RM, Wolf BB, Casiano CA, Newmeyer DD, Ordering the cytochrome c-initiated caspase cascade: hierarchical activation of caspases-2, -3, -6, -7, -8, and -10 in a caspase-9-dependent manner. J Cell Biol. 1999 1 25;144(2):281–92. 9922454 10.1083/jcb.144.2.281 PMC2132895

[11] Zheng TS, Hunot S, Kuida K, Momoi T, Srinivasan A, Nicholson DW, Deficiency in caspase-9 or caspase-3 induces compensatory caspase activation. Nature Medicine. 2000 11;6(11):1241–7. 10.1038/81343 11062535

[12] Simon DJ, Weimer RM, McLaughlin T, Kallop D, Stanger K, Yang J, A Caspase Cascade Regulating Developmental Axon Degeneration. J Neurosci. 2012 12 5;32(49):l7540–53. 10.1523/JNEUROSCI.3012-12.2012 PMC3532512 23223278

[13] Allsopp TE, McLuckie J, Kerr LE, Macleod M, Sharkey J, Kelly JS. Caspase 6 activity initiates caspase 3 activation in cerebellar granule cell apoptosis. Cell Death Differ. 2000 10;7(10) 1984–93. 10.1038/sj.cdd.4400733 11279545

[14] Klaiman G, Champagne N, LeBlanc AC. Self-activation of Caspase-6 in vitro and in vivo: Caspase-6 activation does not induce cell death in HEK293T cells. Biochim Biophys Acta. 2009 3;1793(3):592–601. 19133298 10.1016/j.bbamcr.2008.12.004

[15] Wang XJ, Cao Q, Liu X, Wang KT, Mi W, Zhang Y, Crystal structures of human caspase 6 reveal a new mechanism for intramolecular cleavage self-activation. EMBO Rep. 2010 11;11(11):841–7. 20890311 10.1038/embor.2010.141 PMC2966951

[16] Aharonyl Ehrnhoefer DE, Shruster A QiuX, Franciosi S Hayden MR, A Huntingtin-based peptide inhibitor of caspase-6 provides protection from mutant Huntingtin-induced motor and behavioral deficits. Hum Mol Genet. 2015 5 1;24(9):2604–14. 25616965 10.1093/hmg/ddv023 PMC4383866

[17] Graham RK, Deng Y, Carroll J, Vaid K, Cowan C, Pouladi MA, Cleavage at the 586 amino acid caspase-6 site in mutant huntingtin influences caspase-6 activation in vivo. J Neurosci. 2010 11 10;30(45):15019–29. 21068307 10.1523/JNEUROSCI.2071-10.2010 PMC3074336

[18] Wong BKY, Ehrnhoefer DE, Graham RK, Martin DDO, Ladha S, Uribe V, Partial rescue of some features of Huntington Disease in the genetic absence of caspase-6 in YAC128 mice. Neurobiol Dis. 2015 4;76:24–36. 25583186 10.1016/j.nbd.2014.12.030

[19] Graham RK, Deng Y, Slow EJ, Haigh B, Bissada N, Lu G, Cleavage at the caspase-6 site is required for neuronal dysfunction and degeneration due to mutant huntingtin. Cell. 2006 6 16;125(6):1179–91. 16777606 10.1016/j.cell.2006.04.026

[20] Albrecht S, Bourdeau M, Bennett D, Mufson EJ, Bhattachaijee M, LeBlanc AC. Activation of caspase-6 in aging and mild cognitive impairment. Am J Pathol. 2007 4;170(4):1200–9. 17392160 10.2353/ajpath.2007.060974 PMC1829454

[21] Guo H, Albrecht S, Bourdeau M, Petzke T, Bergeron C, LeBlanc AC. Active caspase-6 and caspase-6-cleaved tau in neuropil threads, neuritic plaques, and neurofibrillary tangles of Alzheimer’s disease. Am J Pathol. 2004 8;165(2):523–31. 15277226 10.1016/S0002-9440(10)63317-2 PMC1618555

[22] LeBlanc AC. Caspase-6 as a novel early target in the treatment of Alzheimer’s disease. Eur J Neurosci. 2013 6;37(12):2005–18. 23773070 10.1111/ejn.12250

[23] Giaime E, Sunyach C, Druon C, Scarzello S, Robert G, Grosso S, Loss of function of DJ-i triggered by Parkinson’s disease-associated mutation is due to proteolytic resistance to caspase-6. Cell Death Differ. 2010 1;17(1):158–69. 19680261 10.1038/cdd.2009.116 PMC2796338

[24] Kobayashi H, Nolan A, Naveed B, Hoshino Y, Segal LN, Fujita Y, Neutrophils Activate Alveolar Macrophages by producing Caspase-6 Mediated Cleavage of Interleukin-1 Associated Kinase-M (IRAK-M). J Immunol. 2011 1 1;186(1):403–10. 21098228 10.4049/jimmunol.1001906 PMC3151149

[25] Berta T, Park C-K, Xu Z-Z, Xie R-G, Liu T, Lü N, Extracellular caspase-6 drives murine inflammatory pain via microglial TNF-α secretion. J Clin Invest. 2014 3;124(3):1173–86. 24531553 10.1172/JCI72230 PMC3934175

[26] Yao Y, Shi Q, Chen B, Wang Q, Li X, Li L, Identification of Caspase-6 as a New Regulator of Alternatively Activated Macrophages. J Biol Chem. 2016 8 12;291(33):17450–66. 27325699 10.1074/jbc.M116.717868 PMC5016141

[27] Zheng M, Karki R, Vogel P, Kanneganti T-D. Caspase-6 Is a Key Regulator of Innate Immunity, Inflammasome Activation, and Host Defense. Cell. 2020 4 15;181(3):674–87. 32298652 10.1016/j.cell.2020.03.040 PMC7425208

[28] Kuriakose T, Man SM, Malireddi RK, Karki R, Kesavardhana S, Place DE, ZBP1/DAI is an innate sensor of influenza virus triggering the NLRP3 inflammasome and programmed cell death pathways. Sci Immunol. 2016 8 5;1(2). 10.1126/sciimmunol.aag2045 PMC5131924 27917412

[29] Kesavardhana S, Malireddi RKS, Burton AR, Porter SN, Vogel P, Pruett-Miller SM, The Zβ2 domain of ZBP1 is a molecular switch regulating influenza-induced PANoptosis and perinatal lethality during development. J Biol Chem. 2020 6 12;295(24):8325–30. 32350114 10.1074/jbc.RA120.013752 PMC7294087

[30] Kesavardhana S, Kuriakose T, Guy CS, Samir P, Malireddi RKS, Mishra A, ZBP1/DAI ubiquitination and sensing of influenza vRNPs activate programmed cell death. J Exp Med. 2017 8 7;214(8):2217–29. 28634194 10.1084/jem.20170550 PMC5551577

[31] Christgen S, Zheng M, Kesavardhana S, Karki R, Malireddi RKS, Banoth B, Identification of the PANoptosome: A Molecular Platform Triggering Pyroptosis, Apoptosis, and Necroptosis (PANoptosis). Front Cell Infect Microbiol. 2020;10:237. 32547960 10.3389/fcimb.2020.00237 PMC7274033

[32] Samir P, Malireddi RKS, Kanneganti T-D. The PANoptosome: A Deadly Protein Complex Driving Pyroptosis, Apoptosis, and Necroptosis (PANoptosis). Front Cell Infect Microbiol. 2020; 10:238. 32582562 10.3389/fcimb.2020.00238 PMC7283380

[33] Zheng M, Kanneganti T-D. The regulation of the ZBP1-NLRP3 inflammasome and its implications in pyroptosis, apoptosis, and necroptosis (PANoptosis). Immunological Reviews. 2020;297(1):26–38. 32729116 10.1111/imr.12909 PMC7811275

[34] Malireddi RKS, Gurung P, Kesavardhana S, Samir P, Burton A, Mummareddy H, Innate immune priming in the absence of TAK1 drives RIPK1 kinase activity-independent pyroptosis, apoptosis, necroptosis, and inflammatory disease. J Exp Med. 2020 3 2;217(3). 10.1084/jem.20191644 PMC7062518 31869420

[35] Gurung P, Burton A, Kanneganti T-D. NLRP3 inflammasome plays a redundant role with caspase 8 to promote IL-1β-mediated osteomyelitis. Proc Natl Acad Sci USA. 2016 4 19;113(10):4452–7. 27071119 10.1073/pnas.1601636113 PMC4843439

[36] Lukens JR, Gurung P, Vogel P, Johnson GR, Carter RA, McGoldrick DJ, Dietary modulation of the microbiome affects autoinflammatory disease. Nature. 2014 12;516(7530):246–9. 25274309 10.1038/nature13788 PMC4268032

[37] Malireddi RKS, Gurung P, Mavuluri J, Dasari TK, Klco JM, Chi H, TAK1 restricts spontaneous NLRP3 activation and cell death to control myeloid proliferation. Journal of Experimental Medicine. 2018 4 2;215(4):1023–34. 10.1084/jem.20171922 PMC5881469 29500178

[38] Zheng M, Williams EP, Malireddi RKS, Karki R, Banoth B, Burton A, Impaired NLRP3 inflammasome activation/pyroptosis leads to robust inflammatory cell death via caspase-8/RIPK3 during coronavirus infection. J Biol Chem. 2020 8 6; 10.1074/jbc.RA120.015036 PMC7549031 32763970

[39] Karki R, Sharma BR, Lee E, Banoth B, Malireddi RKS, Samir P, Interferon regulatory factor 1 regulates PANoptosis to prevent colorectal cancer. JCI Insight. 2020 6 18;5(12). 10.1172/jci.insight.136720 PMC7406299 32554929

[40] Kaiser WJ, Upton JW, Mocarski ES. Receptor-Interacting Protein Homotypic Interaction Motif-Dependent Control of NF-κB Activation via the DNA-Dependent Activator of IFN Regulatory Factors. J Immunol. 2008 11 1; 181(9):0427–34. 10.4049/jimmunol.181.9.6427 PMC3104927 18941233

[41] Nogusa S, Thapa RJ, Dillon CP, Liedmann S, Oguin TH, Ingram JP, RIPK3 Activates Parallel Pathways of MLKL-Driven Necroptosis and FADD-Mediated Apoptosis to Protect against Influenza A Virus. Cell Host Microbe. 2016 7 13;20(1):13–24. 27321907 10.1016/j.chom.2016.05.011 PMC5026823

[42] Thapa RJ, Ingram JP, Ragan KB, Nogusa S, Boyd DF, Benitez AA, DAI Senses Influenza A Virus Genomic RNA and Activates RIPK3-Dependent Cell Death. Cell Host Microbe. 2016 11 9;20(5):674–81. 27746097 10.1016/j.chom.2016.09.014 PMC5687825

[43] Lin J, Kumari S, Kim C, Van TM, Wachsmuth L, Polykratis A, RIPK1 counteracts ZBP1-mediated necroptosis to inhibit inflammation. Nature. 2016 12 1;540(7631):124–8. 27819681 10.1038/nature20558 PMC5755685

[44] Newton K, Wickliffe KE, Maltzman A, Dugger DL, Strasser A, Pham VC, RIPK1 inhibits ZBP1-driven necroptosis during development. Nature. 2016 12 1;540(7031):129–33. 27819682 10.1038/nature20559

[45] Jiao H, Wachsmuth L, Kumari S, Schwarzer R, Lin J, Eren RO, Z-nucleic-acid sensing triggers ZBP1-dependent necroptosis and inflammation. Nature. 2020 3 25;1–5. 10.1038/s41586-020-2129-8 PMC7279955 32296175

[46] Wang R, Li H, Wu J, Cai Z-Y, Li B, Ni H, Gut stem cell necroptosis by genome instability triggers bowel inflammation. Nature. 2020 3 25;1–5. 10.1038/s41586-020-2127-x 32296174

[47] Dagbay KB, Hardy JA. Multiple proteolytic events in caspase-6 self-activation impact conformations of discrete structural regions. PNAS. 2017 9 19; 114(38):E7977–86. 28864531 10.1073/pnas.1704640114 PMC5617271

[48] Li J, McQuade T, Siemer AB, Napetschnig J, Moriwaki K, Hsiao Y-S, The RIP1/RIP3 necrosome forms a functional amyloid signaling complex required for programmed necrosis. Cell. 2012 7 20;150(2):339–50. 22817896 10.1016/j.cell.2012.06.019 PMC3664196

[49] Kang S, Fernandes-Alnemri T, Rogers C, Mayes L, Wang Y, Dillon C, Caspase-8 scaffolding function and MLKL regulate NLRP3 inflammasome activation downstream of TLR3. Nat Commun. 2015 6 24;6(1):1–15. 10.1038/ncomms8515 PMC4480782 26104484

[50] Henry CM, Martin SJ. Caspase-8 Acts in a Non-enzymatic Role as a Scaffold for Assembly of a Pro-inflammatory “FADDosome” Complex upon TRAIL Stimulation. Molecular Cell. 2017 2 16;65(4):715–729.e5. 28212752 10.1016/j.molcel.2017.01.022

[51] Newton K, Wickliffe KE, Maltzman A, Dugger DL, Reja R, Zhang Y, Activity of caspase-8 determines plasticity between cell death pathways. Nature. 2019;575(7784):679–82. 31723262 10.1038/s41586-019-1752-8

[52] Fritsch M, Gunther SD, Schwarzer R, Albert M-C, Schorn F, Werthenbach JP, Caspase-8 is the molecular switch for apoptosis, necroptosis and pyroptosis. Nature. 2019 11;575(7784):683–7. 31748744 10.1038/s41586-019-1770-6

[53] Pham CL, Shanmugam N, Strange M, O’Carroll A, Brown JW, Sierecki E, Viral M45 and necroptosis-associated proteins form heteromeric amyloid assemblies. EMBO reports. 2019 2 1;20(2):e46518. 30498077 10.15252/embr.201846518 PMC6362354

[54] Wegner KW, Saleh D, Degterev A. Complex Pathologic Roles of RIPK1 and RIPK3: Moving Beyond Necroptosis. Trends Pharmacol Sci. 2017 3;38(3) 1202–25. 10.1016/j.tips.2016.12.005 PMC5325808 28126382

[55] Saleh D, Najjar M, Zelic M, Shah S, Nogusa S, Polykratis A, Kinase activities of RIPK1 and RIPK3 can direct IFNβ synthesis induced by lipopolysaccharide. J Immunol. 2017 6 1;198(11):4435–47. 28461567 10.4049/jimmunol.1601717 PMC5471631

[56] Mompeán M, Li W, Li J, Laage S, Siemer AB, Bozkurt G, The Structure of the Necrosome RIPK1-RIPK3 Core, a Human Hetero-Amyloid Signaling Complex. Cell. 2018 5 17;173(5):1244–1253.e10. 29681455 10.1016/j.cell.2018.03.032 PMC6002806

[57] Buendia B, Santa-Maria A, Courvalin JC. Caspase-dependent proteolysis of integral and peripheral proteins of nuclear membranes and nuclear pore complex proteins during apoptosis. J Cell Sci. 1999 6;112 (Pt 11):1743–53. 10318766 10.1242/jcs.112.11.1743

[58] Zheng TS. Learning from Deficiency: Gene Targeting of Caspases. Madame Curie Bioscience Database [Internet]. Landes Bioscience; 2000–2013.

[59] McComb S, Chan PK, Guinot A, Hartmannsdottir H, Jenni S, Dobay MP, Efficient apoptosis requires feedback amplification of upstream apoptotic signals by effector caspase-3 or -7. Sci Adv. 2019 7 31;5(7). 10.1126/sciadv.aau9433 PMC6669006 31392262

